# Psychotherapy Interventions for Managing Anxiety and Depressive Symptoms in Adult Brain Tumor Patients: A Scoping Review

**DOI:** 10.3389/fonc.2015.00116

**Published:** 2015-05-21

**Authors:** Maria Kangas

**Affiliations:** ^1^Department of Psychology, Centre for Emotional Health, Macquarie University, Sydney, NSW, Australia

**Keywords:** brain tumor, psychological treatment, anxiety, depression, emotional well-being

## Abstract

**Background:**

Adult brain tumor (BT) patients and longer-term survivors are susceptible to experiencing emotional problems, including anxiety and/or depression disorders, which may further compromise their quality-of-life (QOL) and general well-being. The objective of this paper is to review psychological approaches for managing anxiety and depressive symptoms in adult BT patients. A review of psychological interventions comprising mixed samples of oncology patients, and which included BT patients is also evaluated. The review concludes with an overview of a recently developed transdiagnostic psychotherapy program, which was specifically designed to treat anxiety and/or depressive symptoms in adult BT patients.

**Methods:**

Electronic databases (PsycINFO, Medline, Embase, and Cochrane) were searched to identify published studies investigating psychological interventions for managing anxiety and depressive symptoms in adult BT patients. Only four randomized controlled trials (RCTs) were identified.

**Results:**

Only one of the RCTs tested a psychosocial intervention, which was specifically developed for primary BT patients, and which was found to improve QOL including existential well-being as well as reducing depressive symptoms. A second study tested a combined cognitive rehabilitation and problem-solving intervention, although was not found to significantly improve mood or QOL. The remaining two studies tested multidisciplinary psychosocial interventions in heterogeneous samples of cancer patients (included BT patients) with advanced stage disease. Maintenance of QOL was found in both studies, although no secondary gains were found for improvements in mood.

**Conclusion:**

There is a notable paucity of psychological interventions for adult BT patients across the illness trajectory. Further research is required to strengthen the evidence base for psychological interventions in managing anxiety and depressive symptoms, and enhancing the QOL of distressed adults diagnosed with a BT.

## Introduction

Adult individuals diagnosed with a primary brain tumor (BT) represent a unique group of patients on the basis that both benign and malignant tumors are associated with disease and treatment side-effects, and can be life-threatening. Depending on the site and size of the BT, these side-effects can lead to substantial neurocognitive, psychosocial, and behavioral problems (1–3). Importantly, a growing body of studies has found that BT survivors are prone to experiencing a high incidence of psychological problems following their diagnosis. Prevalence rates for depression and anxiety symptoms have been documented to be as high as 62% (1, 4). Given the potential life-threatening nature of this disease, several studies have further found that BT survivors may also be susceptible to clinically elevated acute and posttraumatic stress symptoms (PTSS) (5–7).

Research has further shown that if emotional symptoms are left untreated, they have an unremitting, chronic course hampering quality-of-life (QOL) and productivity [e.g., Ref. (8)]. Importantly, these findings accentuate the importance of providing psychological interventions for adult BT patients in order to manage acute psychological problems, as well as prevent chronic psychopathology and maintain QOL in longer-term survivors.

To date, no published study has evaluated the evidence base of psychosocial interventions suitable for distressed (notably, anxious and/or depressed) adult BT patients. This line of inquiry is important in order to guide clinicians and researchers working with this population. Accordingly, the primary objective of this scoping review is to evaluate published psychological-based clinical trials which were either: (a) specifically tailored for adults diagnosed with a primary BT, or (b) comprised heterogeneous oncology patients including BT patients, in order to assess the efficacy of psychological interventions in managing anxiety and depressive symptoms, as well as maintaining or improving the QOL in adult BT patients including longer-term survivors. Given the infancy of this field, this review will conclude with an overview of a recently developed transdiagnostic psychotherapy program, which was specifically designed to treat anxiety and/or depressive disorders in adult BT patients.

## Methods

### Scoping review

Given this type of review is a relatively new methodological approach, to date, there is no universal definition, or consensus on a definitive methodological procedure on reporting guidelines (9). Whereas some authors have proposed that scoping reviews provide a “descriptive overview” of relevant material without critically evaluating and/or synthesizing evidence across different studies (10), more recently, other authors have indicated the importance of synthesizing and critically evaluating the evidence reviewed [e.g., Ref. (11, 12)]. Indeed, without critical evaluation of the methodology of identified studies, researchers and clinicians are unable to delineate relevant gaps in the field in order to guide further research, clinical practice, and policy guidelines (9, 11). To this end, although Arksey and O’Malley in 2005 (10) published the first methodological framework for scoping reviews, several authors have proposed revisions to this framework (11–13). In particular, Pham and colleagues (9) recommend that researchers utilize the Preferred Reporting Items for Systematic Reviews and Meta-Analyses (PRISMA) (14) as a further guide in reporting results for scoping reviews. Accordingly, for the purposes of the current scoping review, Arksey and O’Malley’s (10) six-stage iterative framework was used in combination with relevant components of the PRISMA framework (14). However, as the 6th step (consulting with relevant stakeholders) is proposed to be optional in Arksey and O’Malley’s framework, this final step was excluded for the purposes of the current review. Moreover, in selecting the research question (i.e., Step 1 of Arskey and O’Malley’s framework), Levac and colleagues (13) further recommend to clearly define the question including target population and health outcomes in order to clarify the specific focus of the review. In line with these recommendations as outlined above, the specific aims of the current scoping review explicitly focused on evaluating psychological-based intervention studies for managing anxiety and/or depressive symptoms and/or improving QOL in adult BT survivors. Given this specific focus, the review outcomes are expected to identify relevant gaps in the literature concerning interventions for managing anxiety and depressive symptoms in adult BT patients, in order to guide clinical practice and relevant future research in this area.

### Search strategy

The following electronic databases were searched from their respective inceptions through to the 10 December, 2014: Cochrane, Embase, Ovid Medline, and PsycINFO. The searches were conducted using the following subject headings and/or keywords and combinations: (i) brain (or CNS) tumor/tumour, brain neoplasm, brain cancer, neurooncology, glioma, meningioma; and (ii) psychosocial intervention terms [including counseling/counselling, psychotherapy, psychoeducation, psychosocial intervention/psychosocial/psychological therapy/treatment, stress management, cognitive behavioral/(behavioural) therapy, cognitive therapy, CBT, behaviour/behavior therapy, relaxation]. The searches were restricted to abstracts and articles published in English. A further search was conducted to identify review articles based on psychosocial interventions for oncology populations (specifically comprising heterogeneous samples of oncology patients). The bibliographies of retrieved articles, narrative reviews, and commentary articles on BTs and psychosocial intervention reviews for cancer patients (comprising heterogeneous samples) were also manually searched for additional references. The abstracts of articles identified by electronic searches (1904 in total) as well as manual searches were screened for consideration of inclusion in this review.

### Study selection

All abstracts and/or titles of articles that were identified via electronic and manual searches were screened applying the following selection criteria: (i) published in a peer reviewed journal in full manuscript format; (ii) written in English language; (iii) included a psychosocial intervention (with details of key components provided), which was compared to a standard care, wait-list control, or other type of comparison condition, single design and/or case studies focusing on managing psychological distress were also considered; (iv) participants were a minimum of 18 years of age; (v) the study sample explicitly comprised of patients diagnosed with a primary BT, or for mixed oncology samples, a minimum of 10% of the sample was diagnosed with a BT; and (vi) a specific quantitative measure of psychological well-being (including anxiety and/or depression/mood and/or a QOL scale) was included as a primary or secondary outcome measure, and the measure was administered at minimum pre- and post-intervention.

### Data extraction and evaluation of clinical trials

Studies which met inclusion criteria were read in full, and the following data were extracted and summarized in table format: references, country of study, aim of study, study design, participant characteristics (including sample size, mean age, and BT composition), intervention details, assessment phases and measures, study outcomes, and limitations.

## Results

A total of five published articles (15–19) met the inclusion criteria, although only four of the studies (15–18) were included in this review. The fifth study (19) was excluded, as it was based on the same dataset as one of the included studies (17), and did not report any new data. Although two further case studies were identified (20, 21), they were excluded as both studies did not include a measure of psychological well-being. These two excluded studies focused on testing psychological interventions for managing challenging behaviors in BT patients (20), and managing anger disturbances more broadly with brain injured adults including BT patients (21). In addition, a further recent non-randomized, controlled trial was identified (22), which was designed to evaluate the effectiveness of a multidisciplinary rehabilitation (MDR) program for adults diagnosed with a primary glioma in an Australian community cohort. However, this study was excluded as no details were provided pertaining to the psychological intervention(s) used. Notably, the authors reported that part of the MDR program comprised 30-min blocks of therapy sessions conducted 2–3 times per week for up to 8 weeks, which included psychology, social, occupational, and physiotherapy. However, no specific information was provided as to what these components comprised, and whether the psychological therapy approach was consistent across patients.

Table 1 summarizes the sample characteristics, intervention details, and outcomes of four randomized controlled trials (RCTs). Only two of the four identified studies were solely based on a primary BT sample. Specifically, based on a RCT design, Ownsworth et al. (15) tested the efficacy of a multimodal, home-based intervention [i.e., the “Making Sense of Brain Tumor” (MSoBT) program], which was designed to improve QOL, existential, and mental health well-being in adults with a primary BT. The manualized intervention was administered in individual and/or combined couple/family support sessions, over 10 weekly, 1-h sessions. At post-intervention, patients who received the MSoBT program were found to report significantly lower depressive symptoms, and an improvement in existential and functional well-being relative to patients randomized to a wait-list condition. At 6-months follow-up, the results for the two conditions were combined, and the findings indicated that participants experienced an improvement in existential well-being and overall QOL, as well as a significant decline in depressive symptoms, but not anxiety symptoms.

**Table 1:** **Study characteristics and outcomes**.

Reference and country	Participant characteristics	Intervention design and details	Assessment phases and outcome measures	Results	Limitations
**(A) STUDIES COMPRISING SOLELY OF PRIMARY BRAIN TUMOR PATIENTS**					
Ownsworth et al. (15) Australia	*N* = 50 adults (54% males) diagnosed with primary BT assessed at baseline (min. 18yrs old; range: 17–82) 54% with benign or low grade BTs Mean = 2.6 yrs since BT diagnosis (range: 6 weeks to 18 yrs) *N* = 27 randomized to MSoBT [Ns per Ax: 27, 25, 21]; dropouts due to death (*n* = 2); declined FU assessment (*n* = 3); unable to contact (*n* = 1) *N* = 23 randomized to wait-list control [Ns per Ax: 23, 21, 15]; dropouts due to death (*n* = 2); declined FU assessment (*n* = 2); unable to contact (*n* = 1); withdrew from study before intervention (*n* = 1) or FU assessment (*n* = 2)	RCT with wait-list control MSoBT: 10 × 1 hourly weekly sessions, comprising core components covered in sessions 1, 2, and 10, and module components covering goals, life situation, and cognitive capacity; Program included family members – MSoBT format: home based, manualized, individual and/or combined couple/family support sessions – Tx modules included psycho-education, neuropsychological feedback, cognitive rehabilitation, psychotherapy for anxiety, anger, and depression, couple and family support Wait-list control – received MSoBT after T2 Ax	Ax phases: T1 – baseline T2-Post-Tx T3-6 months FU Measures: – Neuropsych battery to index global functioning – QOL: McGill Quality of Life Questionnaire (MQOL) [inc. existential subscale] – QOL for BT: Functional Assessment of Cancer Therapy – Brain subscale (FACT-BR) (T1 and T2 only) – Depression severity: Montgomery-Asberg Depression Rating Scale (MADRS) – Anxiety, stress, depressive symptoms: Depression Anxiety Stress Scale (DASS21)	All analyses based on *N* = 50, as ITT analyses were used, although only *N* = 44 completed T3 T1–T2 outcomes MSoBT: sig. lower depressive symptoms on MADRS but not on DASS21 MSoBT: sig. elevated existential wellbeing (MQOL) MSoBT: sig. greater functional well-being and total QOL score on FACT-BR, but not for overall score on MQOL T1–T3 outcomes: Sig. lower depressive symptoms on MADRS and DASS21 Sig. higher existential well-being scores and overall QOL (MQOL) Sig. lower stress symptoms on DASS21, but not for anxiety symptoms Secondary analyses at T3: Benign BTs: sig. less depressive symptoms (MADRS) and stress (DASS21), but no sig change for existential well-being, anxiety, or overall QOL (MQOL) Malignant BTs: sig. less depressive symptoms (MADRS) and sig. increase in existential well-being and QOL (MQOL). However, no sig. change on DASS21 scores	No blinding of assessors or therapists, and no details of therapist fidelity checks reported T3/6 mth FU analyses not based on RCT as data merged for both MSoBT and wait-list conditions. Also access to other services between T2 and T3 not monitored

Locke et al. (16) USA	*N* = 19 patient-carer dyads (58% males) diagnosed with a primary BT scheduled to receive radiotherapy (min. 18 yrs old; range: 30–78) 32% with benign or low grade BT 53% also received neurosurgery and 63% received chemotherapy *N* = 9 randomized to the combined intervention plus *N* = 3 directly allocated to the intervention (combined *N* = 12) [*N* = 8 completed study inclusive of 3-mth FU; dropouts due to carer not willing to accompany patient to intervention and fatigue] *N* = 7 randomized to the standard medical care/control condition [*N* = 5 completed 3-mth FU; dropouts due to BT progression]	RCT (quasi-design as final 3 dyads directly allocated to the intervention) with a standard medical care (control) condition Combined Cognitive Rehabilitation (CR) and Problem-Solving (PS) intervention: Each component (CR & PS) comprised 6 × 50 min sessions each over a 2-week period, concurrent with radiotherapy CR component based on Sohlberg and Mateer’s techniques based on using a specific calendar format to compensate for cognitive symptoms PS component was based on Nezu et al.’s techniques and focused on positive problem-solving skills to manage stress reactions Standard medical care (control) condition – no details reported	Ax phases: T1 = baseline T2 = post-Tx T3 = 3 mths FU Primary measures: – Compensation Techniques Questionnaire – Mayo-Portland Adaptability Inventory-4 (MPAI-4) – functional capacity Ax – QOL for BT: Functional Assessment of Cancer Therapy – Brain subscale (FACT-BR) Secondary measures: – The Repeatable Battery for the Assessment of Neuropsychological Status (R-Bans) – test multiple areas of cognitive functioning – One-item Linear Analog Self-Assessment (LASA) – assess overall QOL – The Caregiver QOL Index-Cancer – Profile of Moods State (POMS) – mood severity – The Brief Fatigue Inventory (BFI)	No significant between or within group differences found on any primary or secondary measure	Very small sample size, and low response rate from potential pool of *N* = 160 patients. 38% screened excluded as they had no cognitive deficit, while 16% declined the neuropsych. Ax Patients not recruited on basis of baseline emotional and general well-being, hence results indicate potential ceiling effects as majority of sample were well adjusted emotionally at baseline Due to very low number assessed in person at T3, cognitive Ax could not be conducted No details reported of what the standard medical care condition received and whether access to additional services was assessed
**(B) STUDIES COMPRISING HETEROGENEOUS SAMPLES OF ONCOLOGY PATIENTS INCLUDING BRAIN TUMOR PATIENTS**					
Rummans et al. (17) USA	*N* = 112 adults [*N* = 55 intervention, *N* = 57 standard care] with advanced cancer, diagnosed within last 12 mths, with min. 6 mth survival, and prescribed radiation Tx (min. 2 weeks) Mean age = 59.5 yrs (range: 31–85 yrs) Complete data *N* = 103 (66 males; 12% BT patients) with *N* = 49 intervention (*N* = 6 BTs; 12.2%), and *N* = 54 standard care (*N* = 6 BTS, 11.1%)	RCT with standard care Primary Intervention: – Structured multidisciplinary program comprising 8 × 90 min sessions conducted over 3 weeks – Session structure inc. 20 min physical exercises; educational information; cognitive behavioral strategies for coping with cancer (inc. problem-solving, stress management, assertiveness, relapse prevention); open group discussion (inc. social and spiritual topics, interpersonal relations, grief), and concluded with 10–20 min guided relaxation exercises – Participants issued with workbook Standard care: – Inc. regular medical consults and opportunities for receiving support from outside agencies, e.g., America Cancer Society	Ax phases: T1 – baseline T2-Post-Tx/4 weeks later (primary study end-point) T3-8 weeks from T1 T4–27 weeks from T1 Primary outcome measures: – Spitzer QOL Uniscale & Linear Analog Scales of Assessment of QOL (LASA) Secondary outcome measures: – Symptom Distress Scale – Profile of Mood States (POMS) – Functional Assessment of Chronic Illness Therapy (FACIT) – Spiritual well-being scale	*N* = 103 in final analyses; [*N* = 49 Intervention (*n* = 6 withdrew due to incomplete session attendance), *N* = 54 Standard care (*n* = 3 declined)] T1 to T2: Greater QOL scores for intervention condition (intervention condition increased scores by 3 points – vs. standard care condition decreased scores by 9 points at T2); hence intervention group maintained QOL by T2 T1–T4 (5 mths FU): No sig. group differences, both conditions continued to increase QOL scores Secondary measures: Only POMS – tension/anxiety and confusion/bewilderment subscales improved for the intervention condition at T2	Program limited to participants receiving radiation Tx At post-assessment, 92% (*N* = 45) of intervention grp still receiving radiation Tx at 4 weeks, and 87% (*N* = 47) of standard care grp still receiving radiation Tx Cost of intervention was $2000 per patient for eight sessions Standard care could access outside services

Clark et al. (18) USA	*N* = 131 adults (inc. 22% BTs [*N* = 65 Intervention – inc. *n* = 11 BTs, *N* = 66 standard care – inc. *n* = 18 BTs] with advanced cancer, diagnosed within last 12 mths, with intermediate to poor prognosis and prescribed radiation Tx (min. 1 week) Mean age = 59.3 yrs	RCT with standard care Primary intervention: – Structured multidisciplinary program comprising 6 × 90 min sessions followed by 10 brief structured phone counseling sessions – Session structure inc. 20 min physical exercises; education; cognitive-behavioral strategies; open discussion; support; and concluding with 15 min deep breathing and guided imagery relaxation – Content of program derived from previous Rumman [10] study with several modifications inc. caregiver participation (Sessions 1, 3, 4, and 6); and focus on substance use, mood, anxiety and sleep disorders – Sessions led by clinical psychologist or psychiatrist – Participants issued with 200 page manual Standard care condition: – Received standard medical care services	Ax phases: T1 – baseline T2-Post-Tx/4 weeks later (Primary study end-point) T3 – 27 weeks from T1 Primary outcome measures: – QOL: Functional Assessment of Cancer Therapy (FACT-G) Caregiver QOL: The Caregiver Quality of Life Index – Cancer Scale Secondary outcome measures: – Mood: Profile of Mood States (POMS) – Functional Assessment of Chronic Illness Therapy (FACIT) – Spiritual well-being scale – Sleep: Pittsburgh Sleep Quality Index – Exercise behaviors	T1–T2 Complete data at primary endpoint (week 4 post-baseline) *N* = 117 [*N* = 54 intervention; *N* = 63 standard care]; drop-outs due to no baseline and/or T2 data (*n* = 10); death (*n* = 1), incomplete treatment sessions (fout attended) (*n* = 7) – Greater QOL scores for intervention condition (especially physical and functional wellbeing scores) (intervention condition maintained scores – vs. standard care condition decreased scores at T2) T1–T3 (5 mths FU): (*N* = 110; *N* = 51 intervention, *N* = 59 Standard care) – No sig. group differences, both conditions continued to increase QOL scores (back to baseline/T1 levels) Secondary measures: – All measures were not sig at both T2 and T3 Caregiver QOL: No sig differences between conditions	Program limited to participants receiving radiation Tx

*Ax, assessment; BT, brain tumor; grp, group; inc., includes/including; FU, follow-up; min, minimum; mths, months; ITT, intent-to treat; *N*, number of participants; QOL, quality-of-life; RCT, randomized control trial; sig., significant; T1, assessment phase 1 (baseline); T2, assessment phase 2; T3, assessment phase 3; T4, assessment phase 4; Tx, treatment; yrs, years*.

In the second study which focused solely on BT patients, Locke et al. (16) quasi-randomly allocated a very small dyadic sample (*n* = 19) of adults diagnosed with a primary BT and their carers, to a combined cognitive rehabilitation and problem-solving intervention or to the standard medical (control) condition. The design was quasi-RCT as the final three patient-dyads recruited were directly allocated to the intervention condition. The majority of participants (88%, *n* = 7 of 8) who received the intervention reported using the intervention strategies for a minimum, several times per week at the end of the 2-week trial period, while 50% continued to use these strategies several times per week at 3-months follow-up. Similarly, 88% of participants in the intervention condition reported finding the program “somewhat” or “very” helpful. However, no differences were found between the intervention and standard care/control conditions in terms of improvement in QOL, functional capacity, emotional distress, or fatigue at the completion of the trial period, or at the 3-months follow-up assessment. In particular, a high proportion of participants (*n* = 16) reported average to above average scores on the QOL measure at baseline, and at the two subsequent follow-up assessments. Comparably, over 77% of participants mood scores indicated good emotional adjustment at each of the three assessments (inclusive of the baseline period). Furthermore, the effects of the intervention on cognitive functioning could not be tested, as the majority of patients at the follow-up assessment were assessed via telephone, thus ruling out the administration of the neurocognitive test.

The other two randomized controlled studies were based on mixed samples of patients diagnosed with advanced cancer. In the first study, Rummans et al. (17) tested the efficacy of a structured multidisciplinary intervention in maintaining QOL in advanced cancer patients (including 12% of patients with BTs) scheduled to receive a minimum of 2 weeks radiation treatment. The group-based intervention comprised eight weekly, 90 min sessions conducted over 3 weeks, and which included weekly physical and relaxation exercises as well as cognitive-behavioral strategies and open group discussions. The primary endpoint was 4 weeks post-baseline assessment (i.e., 1 week post-intervention), although a 27-week (5-month) follow-up assessment was also conducted. The results revealed that at 1-week post-intervention, participants were found to maintain their QOL relative to patients randomized to the standard care condition, which reported a decline in QOL scores. However, by 5 months follow-up, no significant differences were evident between conditions. Specifically, both groups QOL scores were comparable to baseline functioning.

In a more recent study, Clark et al. (18) adapted Rumman et al.’s (17) program and tested the efficacy of this structured multidisciplinary intervention in maintaining QOL in a further sample of advanced cancer patients (including 22% with BTs), which were recommended to receive at least 1-week of radiation treatment. In this study, the intervention was reduced to six sessions, which included caregiver participation, as well as an additional 10 brief follow-up phone counseling sessions. The primary endpoint for this study was also at 4 weeks following the baseline assessment, although a 5-month follow-up was also included. At the 4-week assessment, participants in the intervention condition reported elevated QOL scores, particularly an improvement in physical well-being, relative to participants in standard care who reported a decline in QOL scores. However, by 5-months follow-up, no significant differences were evident between conditions. Notably, both groups had returned to baseline functioning for QOL outcomes. Additionally, no significant group differences emerged at 4-week or 5-months follow-up for the secondary measures including emotional and spiritual well-being, as well as sleep functioning.

## Discussion

For this scoping review, only four published RCT studies (15–18) [one of which was a quasi-RCT design; (16)] were identified, which included adult BT patients in evaluating the efficacy of psychosocial interventions designed to maintain and/or improve QOL including existential well-being. Three of the studies (15, 17, 18) comprised structured, manualized multimodal interventions, which included cognitive-behavioral strategies. The fourth study (16) comprised a quasi-randomized design given the final 3 patient dyads were directly allocated to the intervention condition, which comprised a combined cognitive rehabilitation and structured problem-solving therapy program.

In one of the only two published studies to date, which were specifically designed for adults diagnosed with primary BTs, Ownsworth et al. (15) found that the MSoBT program was found to improve QOL and existential well-being, and reduce depressive symptoms up to 6-months post-intervention. Given this is the only published RCT study, which was designed for BT patients, this reflects the notable paucity of psychosocial interventions specifically tailored for adult BT patients. Although the findings from this study are promising, there are several short-comings that need to be considered in informing future interventions in this field. First, only the initial, post-treatment results were based on the RCT design, as the 6-month follow-up outcomes included participants allocated to the wait-list condition. Second, the intervention was not found to improve anxiety symptoms. Furthermore, although depressive symptoms were found to improve post-intervention, this study was not specifically tailored to clinically distressed (i.e., anxious and/or depressed) BT patients. Hence, the effect of this intervention in managing anxiety and depressive disorders in distressed BT patients is unknown. Third, given the multimodal intervention, which also included neuropsychological feedback and cognitive rehabilitation, it is not clear which components contributed to specific treatment gains. Finally, the home-based, in-person therapy sessions raise feasibility issues for hospital and community settings, which may not have the resources to roster staff to weekly offsite home visits.

In the second study, which was specifically designed for patients with a primary BT (as well as their carers) (16), a combined cognitive rehabilitation and positive problem-solving intervention was found to be acceptable by patients newly diagnosed with BT undergoing radiation treatment, and who were assessed to have mild to moderate cognitive impairments. However, this combined intervention was not found to lead to significant improvements in terms of QOL, functional capacity, mood or fatigue compared to patients who received standard medical care. These null outcomes can most likely be attributed to the majority of patients having good emotional adjustment and general well-being at baseline (i.e., soon after their BT diagnosis), and which was maintained at 3-months follow-up. In addition, given the very small sample size (*N* = 19 dyads at baseline and *N* = 13 at the 3-month follow-up), Locke et al. acknowledge that their study was not adequately powered to detect statistical changes. Although the majority of patients who received the combined intervention reported finding this program “somewhat” to “very” helpful, the findings indicate that patients which are relatively well adjusted emotionally may not derive further benefits in terms of improvement of mood and QOL by receiving an intensive cognitive and psychological short-term intervention while undergoing radiation treatment. Indeed, Locke et al. recommend that for future research, targeting BT patients who report reduced QOL, elevated emotional distress, and/or poor functional performance may be fruitful to further test the feasibility of this type of intervention.

The other two RCTs (17, 18) included in this review comprised heterogeneous samples of adults diagnosed with advanced cancer (including BT patients), who were recommended to receive radiation treatment. Both RCTs were based on a comparable structured, multidisciplinary psychosocial intervention, which also included physical and relaxation exercises. Interestingly, both studies were found to facilitate maintenance of QOL within 4 weeks post-baseline (on average 1-week post-intervention). However, the intervention was not found to differ from standard care at 5-months follow-up. A potential explanation for this latter outcome is that participants in standard care were documented to have the opportunity to receive external support from agencies such as the American Cancer Society [e.g., Ref. (17)]. However, access to additional support services was not reported to be monitored in these trials. It cannot therefore be ruled out that the lack of group differences at 5-months follow-up may in part be due to patients in the standard care condition accessing external support. A further reason may also in part be due to the recovery period required to overcome the acute radiation treatment side-effects. In fact, at the 4-week assessment, more than 85% of the sample was still receiving radiation treatment in the Rummans et al. (17) study. Taken together, these findings suggest that this multidisciplinary program is useful in helping patients maintain their QOL during the course of their radiation treatment, although there does not seem to be any additive benefits at 5-months follow-up. This is further reflected in the non-significant results reported for the secondary outcome measures. Notably, the intervention was not found to lead to significant improvements in psychological distress, mood, and spiritual well-being. Moreover, given the heterogeneous sample composition, which comprised only a small proportion of patients diagnosed with BTs (between 12 and 22%), the findings from these two RCTs for patients with advanced BTs are considered preliminary.

Collectively, the findings from this review accentuate the paucity of studies that have been specifically designed for primary BT patients, and which have assessed the effects of psychological interventions in managing anxiety and/or depressive symptoms and/or improving QOL. Although a number of cognitive rehabilitation programs for BT patients have also included measures of psychological distress and/or QOL [e.g., Ref. (23)], however, with the exception of Locke et al.’s combined cognitive and problem-solving intervention (16), no further published cognitive rehabilitation studies were identified that also included a psychological intervention. Moreover, as aforementioned, one controlled MDR program for adult BT patients was identified (22), and which did include access to psychological therapy. However, no details were reported pertaining to what the therapy entailed, and whether this was consistent across patients. Notwithstanding this lack of detail, interestingly, this program was not found to lead to significant improvements in anxiety, depressive, and QOL scores for participants who completed the MDR program. Comparable to Locke et al.’s findings (16), the null results from the MDR program may be due to patients not being clinically distressed at baseline, and hence, did not derive significant improvement in emotional well-being by having access to psychotherapy. Taken together, the findings from these two integrative cognitive and psychological intervention programs attest to the need to conjointly screen for both cognitive impairment and emotional distress to ascertain which patients may benefit most from multidisciplinary interventions which include a psychotherapy component.

Importantly, given the high rates of psychological problems experienced by BT patients [e.g., Ref. (1, 4)], it is surprising that no published study to date was identified which was specifically tailored to clinically distressed BT patients. Indeed, small to medium effects with mixed outcomes have emerged in the efficacy of psychosocial interventions in managing emotional disturbances in the broader (non-BT) oncology literature (24, 25). This may in part be due to floor effects if patients are not experiencing clinically elevated levels of distress at baseline. Indeed, in the most recent and largest meta-analytic review of the effects of psychosocial interventions to manage emotional distress and QOL in cancer patients, Faller et al. (24) found that only a very limited number of studies preselected participants according to baseline distress levels. Importantly, these studies showed the largest effect sizes. In the current review, although Ownsworth et al. (15) found a reduction in depressive symptoms, this was not comparably found for anxiety symptoms. Moreover, the clinical diagnostic status of patients at baseline was not reported.

Research has shown that depression and anxiety disorders share common latent structures, which have contributed to a recent increase in studies testing the concurrent treatment of anxiety and mood disorders in the general population using cognitive and behavioral based transdiagnostic therapies (26), including the integration of behavioral strategies with acceptance-based therapies [such as Acceptance and Commitment Therapy (ACT)] (27, 28). Considering that anxiety problems have been found to be associated with comorbid depressive symptoms in adults diagnosed with a primary BT [e.g., Ref. (4, 7)], ACT-based interventions may have particular relevance for distressed BT patients given the objective of this approach is to improve patients functionality and QOL in concordance with their values, while also factoring in their shortcomings (19–30). Hence, for adult BT patients, contingent on the extent of cognitive, sensory, and physical deficits sustained due to the tumor and/or treatment side-effects, patients can still re-learn how to maintain an adaptive QOL by engaging in value-based activities (e.g., enjoyment of participating in team-based social events) they can partake in, despite any impairment(s) they have sustained. Moreover, ACT aligns with transdiagnostic approaches as the treatment components are comparable for both managing anxiety and depressive disturbances.

### An ACT-based transdiagnostic intervention for BT patients: A pilot case study

Kangas et al. (31) developed a manualized ACT-based transdiagnostic intervention (which included patient handouts, worksheets, and a CD) with the aim of improving the emotional well-being and QOL of distressed adults with primary BTs. In line with ACT principles, the program comprised the following components which targeted: (1) education about common reactions to being diagnosed with a BT; (2) acceptance, defusion, and mindfulness based exercises to promote awareness and acceptance of internal physical sensations and cognitions; (3) learning to deal with the uncertainty of a BT diagnosis and prognosis by using acceptance and mindfulness strategies; (4) behavioral exercises to re-engage in avoided activities; and (5) re-evaluating life-goals concordant with one’s values in at least three key domains – interpersonal, personal/self-care, and occupational, in order to engage or re-engage and commit to pursuing valued life goals. This program was designed to be conducted in-person over 6-weekly, 90 min sessions, including two additional “booster” sessions to consolidate skills learnt, scheduled at fortnightly intervals.

Kangas et al. (31) initially pilot tested the ACT intervention with a middle-aged male, “Luke” (aged 53 years), diagnosed with a meningioma 2.3 years prior and had completed his BT treatment (including a craniotomy and 20 sessions of fractionated stereotactic radiotherapy) 18 months prior to referral to the program. Luke completed a comprehensive assessment including a diagnostic clinical interview, self-report measures, and neuropsychological testing at four phases: baseline (T1), end of therapy (T2), 1-month (T3), and 3-months (T4), following completion of the 8-session program. At baseline, Luke met comorbid criteria for both Major Depressive Disorder and anxiety (including Generalized Anxiety Disorder and BT-related PTSD). His QOL scores on the Functional Assessment of Cancer Therapy – General and Brain subscale (FACT-G/BR) (32) were very low, >3 SDs below published norms. He also reported low acceptance and high experiential avoidance of negative cognitions and physiological bodily sensations since his BT diagnosis, as assessed by the Acceptance of Actions Questionnaire (AAQ) (33). This scale measures an individual’s avoidance of negative perceived cognitions (including thoughts, beliefs, and perceptions) and physical sensations. His memory and cognitive skills were assessed to be in the average to above average range, although his problem-solving scores were slightly below average. By the end of therapy, Luke no longer met criteria for depression or anxiety. With a reduction in depressive symptoms [as measured by the Beck Depression Inventory – 2nd Edition; (34)], and a decline in experiential avoidance, Luke also increased his problem-solving skills and QOL. In particular, he engaged in substantially more social events and reintegrated with his social network. These effects were maintained at 3-months follow-up. These case report findings demonstrate that this ACT-based intervention has potential, promising utility in treating both anxiety and depression disturbances in distressed adults diagnosed and treated for a primary BT. However, the efficacy of this program needs to be tested in future research using a large scale controlled trial design, particularly as there is a dearth of studies that have been specifically tailored for distressed adult BT survivors.

It is acknowledged that this scoping review was limited to published studies in the English language. Also, the abstracts and extracted data were not independently evaluated by a second reviewer. Notwithstanding these limitations, a more integrative scoping review method was used to keep the study aim specifically focused on identifying psychological interventions for adult BT patients which anxiety, depression, and/or QOL indices were included as outcome measures in evaluating the effects of the intervention. Moreover, in accord with recent recommendations for scoping reviews (9), the methodology of identified studies was critically evaluated in line with relevant components of the PRISMA (14) framework in order to clearly delineate gaps in this field and guide future research trials.

## Conclusion

In conclusion, the findings from this scoping review demonstrate that there is a notable paucity of published controlled trials which have tested the efficacy of any type of psychological based intervention in managing the emotional wellbeing (notably, anxiety and/or depressive symptoms) and QOL of adults diagnosed with a primary BT across the illness trajectory. To date, the outcome from Ownsworth et al.’s (15) RCT study accentuates the potential utility in using a multimodal approach including cognitive and behavioral strategies to enhance the QOL and existential wellbeing of BT survivors. In order to strengthen the evidence base in this field, future research is pressingly warranted to further test the efficacy of psychological interventions in managing emotional disturbances and maintaining and/or improving the QOL, particularly in clinically distressed BT patients and longer-term survivors.

## Conflict of Interest Statement

The author declares that the research was conducted in the absence of any commercial or financial relationships that could be construed as a potential conflict of interest.
